# Lower circulating neuron-specific enolase concentrations in adults and adolescents with severe mental illness

**DOI:** 10.1017/S0033291721003056

**Published:** 2023-03

**Authors:** Dimitrios Andreou, Nils Eiel Steen, Kjetil Nordbø Jørgensen, Runar Elle Smelror, Kirsten Wedervang-Resell, Stener Nerland, Lars T. Westlye, Terje Nærland, Anne Margrethe Myhre, Inge Joa, Solveig Merete Klæbo Reitan, Arne Vaaler, Gunnar Morken, Erlend Bøen, Torbjørn Elvsåshagen, Birgitte Boye, Ulrik Fredrik Malt, Pål Aukrust, Silje Skrede, Rune Andreas Kroken, Erik Johnsen, Srdjan Djurovic, Ole A. Andreassen, Thor Ueland, Ingrid Agartz

**Affiliations:** 1 Norwegian Centre for Mental Disorders Research (NORMENT), Institute of Clinical Medicine, University of Oslo, Oslo, Norway; 2 Department of Clinical Neuroscience, Centre for Psychiatry Research, Karolinska Institutet & Stockholm Health Care Services, Stockholm County Council, Stockholm, Sweden; 3 Division of Mental Health and Addiction, Norwegian Centre for Mental Disorders Research (NORMENT), Oslo University Hospital, Oslo, Norway; 4 Department of Psychiatric Research, Diakonhjemmet Hospital, Oslo, Norway; 5 Child and Adolescent Mental Health Research Unit, Division of Mental Health and Addiction, Department of Research and Innovation, Oslo University Hospital, Oslo, Norway; 6 Department of Psychology, University of Oslo, Oslo, Norway; 7 K.G. Jebsen Center for Neurodevelopmental Disorders, Institute of Clinical Medicine, University of Oslo, Oslo, Norway; 8 NevSom, Department of Rare Disorders, Oslo University Hospital, Oslo, Norway; 9 Division of Mental Health and Addiction, Department of Research and Innovation, Oslo University Hospital, Oslo, Norway; 10 TIPS – Network for Clinical Research in Psychosis, Stavanger University Hospital, Stavanger, Norway; 11 Faculty of Health, Network for Medical Sciences, University of Stavanger, Stavanger, Norway; 12 Faculty of Medicine and Health Sciences, Department of Mental Health, NTNU, Trondheim, Norway; 13 St Olavs Hospital, Department of Mental Health, Trondheim, Norway; 14 Psychosomatic and C-L Psychiatry, Adult, Division of Mental Health and Addiction, Oslo University Hospital, Oslo, Norway; 15 Department of Neurology, Division of Clinical Neuroscience, Oslo University Hospital, Oslo, Norway; 16 Institute of Clinical Medicine, University of Oslo, Oslo, Norway; 17 Department of Behavioural Medicine, University of Oslo, Oslo, Norway; 18 Research Institute of Internal Medicine, Section of Clinical Immunology and Infectious Diseases, Oslo University Hospital, Rikshospitalet, Oslo, Norway; 19 Department of Clinical Science, University of Bergen, Bergen, Norway; 20 Department of Medical Biochemistry and Pharmacology, Haukeland University Hospital, Bergen, Norway; 21 Division of Psychiatry, Haukeland University Hospital, Bergen, Norway; 22 Department of Clinical Medicine, University of Bergen, Bergen, Norway; 23 Norwegian Centre for Mental Disorders Research (NORMENT), Haukeland University Hospital, Bergen, Norway; 24 Department of Medical Genetics, Oslo University Hospital, Oslo, Norway; 25 Department of Clinical Science, Norwegian Centre for Mental Disorders Research (NORMENT), University of Bergen, Bergen, Norway; 26 K.G. Jebsen Thrombosis Research and Expertise Center, University of Tromsø, Tromsø, Norway; 27 Research Institute of Internal Medicine, Oslo University Hospital, Rikshospitalet, Oslo, Norway

**Keywords:** Bipolar disorder, ENO2, schizophrenia, total grey matter volume

## Abstract

**Background:**

Both neurodegenerative and neurodevelopmental abnormalities have been suggested to be part of the etiopathology of severe mental illness (SMI). Neuron-specific enolase (NSE), mainly located in the neuronal cytoplasm, may indicate the process as it is upregulated after neuronal injury while a switch from non-neuronal enolase to NSE occurs during neuronal maturation.

**Methods:**

We included 1132 adult patients with SMI [schizophrenia (SZ) or bipolar spectrum disorders], 903 adult healthy controls (HC), 32 adolescent patients with SMI and 67 adolescent HC. Plasma NSE concentrations were measured by enzyme immunoassay. For 842 adults and 85 adolescents, we used total grey matter volume (TGMV) based on T1-weighted magnetic resonance images processed in FreeSurfer v6.0. We explored NSE case-control differences in adults and adolescents separately. To investigate whether putative case-control differences in NSE were TGMV-dependent we controlled for TGMV.

**Results:**

We found significantly lower NSE concentrations in both adult (*p* < 0.001) and adolescent patients with SMI (*p* = 0.007) compared to HC. The results remained significant after controlling for TGMV. Among adults, both patients with SZ spectrum (*p* < 0.001) and bipolar spectrum disorders (*p* = 0.005) had lower NSE than HC. In both patient subgroups, lower NSE levels were associated with increased symptom severity. Among adults (*p* < 0.001) and adolescents (*p* = 0.040), females had lower NSE concentrations than males.

**Conclusion:**

We found lower NSE concentrations in adult and adolescent patients with SMI compared to HC. The results suggest the lack of progressive neuronal injury, and may reflect abnormal neuronal maturation. This provides further support of a neurodevelopmental rather than a neurodegenerative mechanism in SMI.

## Introduction

Schizophrenia (SZ) and bipolar disorder (BD) are severe mental illnesses (SMIs) each affecting approximately 1% of the population (Kahn et al., 2015; Vieta et al., 2018). The disorders are leading causes of disability, have a complex and largely unknown etiopathology (Kahn et al., 2015; Kloiber et al., 2020), are highly heritable (Mullins et al., 2021; Smeland, Frei, Dale, & Andreassen, 2020) and stigmatised (Hawke, Parikh, & Michalak, 2013; Serafini et al., 2011; Valery & Prouteau, 2020). Several lines of evidence indicate neurodevelopmental abnormalities in SMI (Kinros, Reichenberg, & Frangou, 2010; O'Shea & McInnis, 2016; Owen, O'Donovan, Thapar, & Craddock, 2011). Furthermore, there are indications of enhanced neurodegeneration with increasing symptom load over time, cognitive decline and progressive brain changes (Goodwin, Martinez-Aran, Glahn, & Vieta, 2008; Lieberman, 1999; Vieta et al., 2018).

Enolases are enzymes that participate in glycolysis and gluconeogenesis. In particular, enolases catalyse the conversion of 2-phosphoglycerate to phosphoenolpyruvate in glycolysis, and the reverse reaction in gluconeogenesis (Isgro, Bottoni, & Scatena, 2015). In vertebrates, there are three dimeric isoforms: the non-neuronal enolase (NNE) or enolase 1, the neuron-specific enolase (NSE) or enolase 2, and the muscle-specific enolase or enolase 3 (Haque, Polcyn, Matzelle, & Banik, 2018). In the central nervous system, both NSE and NNE are expressed (Haque et al., 2018; Isgro et al., 2015). NSE, first described in 1965 (Moore & McGregor, 1965), is a soluble protein present mainly in the cytoplasm of neurons, both in the cortex and in subcortical regions, and constitutes a significant fraction of the total soluble protein of the brain (Marangos & Schmechel, 1987).

Increased cerebrospinal fluid (CSF) and circulating (plasma/serum) NSE concentrations indicate neuronal damage (Haque et al., 2018; Isgro et al., 2015). In particular, elevated NSE reflects neuronal damage after traumatic brain injury (Cheng, Yuan, Yang, Wang, & Liu, 2014) and may also indicate oxidative damage underlying neurodegenerative disorders (Haque et al., 2018). Circulating NSE levels were higher in symptomatic Huntington' disease (Ciancarelli et al., 2015), and CSF (Schmidt et al., 2014) but not circulating (Chaves et al., 2010) NSE levels were elevated in Alzheimer's disease, whereas the NSE literature in Parkinsonian syndromes is conflicting (Constantinescu, Zetterberg, Holmberg, & Rosengren, 2009; Schaf et al., 2005). Increased CSF NSE has also been shown in neurological disorders with rapid progression such as Guillain–Barré syndrome (Mokuno et al., 1994) and Creutzfeldt–Jakob disease (Aksamit, Preissner, & Homburger, 2001). If SMIs are neurodegenerative disorders with ongoing neuronal damage, circulating NSE levels might be increased.

NSE is also involved in neuronal differentiation, maturation and migration (Haque et al., 2018; Isgro et al., 2015; Marangos & Schmechel, 1987). In the mammalian embryonic brain and in primary neuron cultures, undifferentiated neurons have been shown to contain mainly NNE, whereas a switch to NSE occurs during the neuronal maturation process and cell migration (Marangos & Schmechel, 1987; Marangos, Schmechel, Parma, & Goodwin, 1980b; Schengrund & Marangos, 1980; Schmechel, Brightman, & Marangos, 1980). It has been suggested that the key to the development of psychosis is early (pre- and perinatal) but also late (during adolescence) neurodevelopmental disturbances (Brent, Thermenos, Keshavan, & Seidman, 2013). There is indication of neuronal maturation disturbances in both SZ and BD (Gandal, Nesbitt, McCurdy, & Alter, 2012; Hagihara, Ohira, Takao, & Miyakawa, 2014; Hagihara, Takao, Walton, Matsumoto, & Miyakawa, 2013; Torkamani, Dean, Schork, & Thomas, 2010; Walton et al., 2012). In the prefrontal cortex (PFC) of patients with SZ, the normal age-related decline in gene expression associated with developmental processes, including neuronal differentiation, is slowed (Torkamani et al., 2010). Hagihara et al. have shown a transcriptional immaturity in the PFC of patients with SZ that was not due to medication effects (Hagihara et al., 2014). Further, fast-spiking interneurons, the primary cells for synaptic inhibition, have been suggested to be immature in the cortex of patients with SZ and BD (Gandal et al., 2012). Finally, both animal models and post-mortem studies have indicated an immature hippocampal dentate gyrus endophenotype in SZ and BD, with abundant immature granule cells (Hagihara et al., 2013; Walton et al., 2012). Thus, if circulating NSE reflects such disturbances, one might expect lower NSE levels in SMI than in HC.

Previous studies with small sample sizes have shown unaltered circulating NSE levels in SZ (Egan et al., 1992; Schroeter, Abdul-Khaliq, Krebs, Diefenbacher, & Blasig, 2009; Steiner, Bielau, Bernstein, Bogerts, & Wunderlich, 2006), while studies of circulating NSE in BD have shown conflicting results (Akcan, Karabulut, Ismail Kucukali, Cakir, & Tuzun, 2018; Karabulut et al., 2019; Machado-Vieira et al., 2007; Tsai & Huang, 2017; Wiener et al., 2013). The study of circulating NSE levels in a large-scale context might provide more conclusive answers. Herein, we hypothesise that circulating NSE concentrations in adult and adolescent patients with SMI are either increased (indicating progressive neuronal damage) or decreased (suggesting neural maturation disturbance).

We further aimed to explore whether putative case-control differences in circulating NSE are dependent on total grey matter volume (TGMV). Smaller total or regional grey matter volumes have been reported in SMI (Haijma et al., 2013; Hibar et al., 2016; Wang et al., 2019), even in first-episode psychosis and in patients at high risk for psychosis (Fusar-Poli, Smieskova, Serafini, Politi, & Borgwardt, 2014). As neurons are the main cells producing NSE (Marangos & Schmechel, 1987), it has been hypothesised, but never shown, that smaller grey matter volume is associated with reduced circulating NSE concentrations (Hoffmann et al., 2017). In a population-based study, Hoffmann et al. reported no significant association (Hoffmann et al., 2017), but to the best of our knowledge, this has not been investigated in SMI.

## Materials and methods

### Participants

Patients were recruited from outpatient and inpatient psychiatric units in Norway, as part of the Thematically Organised Psychosis (TOP) research study (adult participants) and the TOP-Study for Youth (Youth-TOP; adolescent participants). Healthy controls (HC) were recruited from the same catchment area as the patients using the national population register. The TOP research study and the Youth-TOP research study are the main study protocols at the Norwegian Centre for Mental Disorders Research (NORMENT, Oslo, Norway; www.med.uio.no/norment/english), for adults and adolescents, respectively. For the current study, participants were drawn from the TOP and the Youth-TOP study cohorts if NSE data were available. Patients were included if they were diagnosed with SZ spectrum or BD spectrum disorders according to DSM-IV as described below. The following exclusion criteria were applied for all participants: previous moderate or severe head injury, neurological disorders or medical conditions that could affect brain function. In addition, to the best of our knowledge, none of the participants had medical conditions associated with increased circulating NSE levels including haemolytic anaemia (Geisen et al., 2015), hepatic failure (Strauss et al., 2001), end-stage renal disease (Davis et al., 2011) or a history of NSE-secreting tumours, i.e. neuroendocrine tumours, lung cancer, mainly small-cell lung cancer, neuroblastoma, melanoma, seminoma or other rare tumours where raised NSE levels have been observed (renal cell carcinoma, carcinoid tumours, dysgerminomas and immature teratomas) (Isgro et al., 2015). HC with previous or current psychiatric disorders including substance use disorder (including alcohol use disorder) or with first-degree relatives with SMI were excluded. Adolescent patients who met the criteria for substance use disorder (including alcohol use disorder) were also excluded.

#### Adult sample (TOP study)

We included 1132 adult patients with SMI and 903 adult HC (age range 18–65 years). We enrolled patients with SZ spectrum disorders (*n* = 735), i.e. patients with SZ (*n* = 424), schizophreniform disorder (*n* = 44), schizoaffective disorder (*n* = 103), delusional disorder (*n* = 45), brief psychotic disorder (*n* = 11), psychotic disorder not otherwise specified (NOS) (*n* = 108), and patients with BD spectrum (*n* = 397), i.e. BD I (*n* = 249), BD II (*n* = 128) and BD NOS (*n* = 20). Medical doctors and psychologists assessed the patients with the Structured Clinical Interview for DSM-IV axis I disorder (SCID-I) module A–E (Spitzer, Williams, Gibbon, & First, 1988). HC were assessed for exclusion criteria including the use of Primary Care Evaluation of Mental Disorders (Prime-MD) (Spitzer et al., 1994).

#### Adolescent sample (Youth-TOP study)

We included 32 adolescent patients with SMI (early-onset psychosis; onset of illness <18 years of age) (Werry, McClellan, & Chard, 1991) and 67 adolescent HC (age range 12–18 years). We enrolled patients with SZ (*n* = 17), schizoaffective disorder (*n* = 1), brief psychotic disorder (*n* = 1), psychotic disorder NOS (*n* = 11) and BD I with psychotic features (*n* = 2), according to the DSM-IV. Medical doctors and psychologists assessed the patients and HC with the Schedule for Affective Disorders and Schizophrenia for School Aged Children-Present and Lifetime Version (K-SADS-PL) (Kaufman et al., 1997).

### Measures and medication

We evaluated the patients with the Positive and Negative Syndrome Scale (PANSS) (Kay, Fiszbein, & Opler, 1987) and the Young Mania Rating Scale (YMRS) (Young, Biggs, Ziegler, & Meyer, 1978). PANSS is a 30-item standardised clinical interview for the assessment of positive (seven items) and negative symptoms (seven items) as well as general psychopathology (16 items) related to psychosis. The PANSS total score ranges from 30 to 210 (positive scale: 7–49, negative scale: 7–49, general psychopathology scale 16–112) with higher scores indicating increased symptom severity (Kay et al., 1987). YMRS is an 11-item interviewer rating scale used to evaluate manic symptoms. The ‘irritability’, ‘speech’, ‘thought content’ and ‘disruptive-aggressive behaviour’ items are double weighted, and the YMRS total score ranges from 0 to 60 with higher scores indicating more severe manic symptoms (Young et al., 1978).

We assessed the current use (yes/no) of antipsychotic, antiepileptic, lithium, and antidepressive medication, and for patients on antipsychotics we calculated the current chlorpromazine equivalent doses (CPZ) in mg/day (Andreasen, Pressler, Nopoulos, Miller, & Ho, 2010). In the adolescent sample we had information on lifetime antipsychotic medication exposure, and calculated lifetime CPZ (CPZ years) (Andreasen et al., 2010). We defined the age of onset as the age at first psychotic episode in SZ spectrum and at first-affective episode in BD spectrum. In adults, we evaluated alcohol use with the alcohol use disorder identification test (AUDIT) (Bohn, Babor, & Kranzler, 1995) and drug use with the drug use disorder identification test (DUDIT) (Berman, Bergman, Palmstierna, & Schlyter, 2005).

### NSE measurement

Plasma levels of NSE were measured in duplicate by enzyme immunoassays (EIA) using commercially available antibodies (R&D Systems, Minneapolis, MN, USA) in a 384 format using a combination of a SELMA (Jena, Germany) pipetting robot and a BioTek (Winooski, VT, USA) dispenser/washer. Absorption was read at 450 nm with wavelength correction set to 540 nm using an ELISA plate reader (Bio-Rad, Hercules, CA, USA). Blood was sampled on ethylenediaminetetraacetic acid vials, and the plasma was isolated and stored at −80°C. Blood sampling was performed between 8 am and 5 pm with some variation between patients and HC. We found no effect of diurnal variation comparing non-fasting levels in individuals at 8 am and 4 pm [*n* = 6, within-patient coefficient of variation (CV) = 16%, *p* = 0.43] or postprandial variation comparing fasting and non-fasting samples at 8 am (*n* = 6, within-patient CV = 12%, *p* = 0.38). Validation of the NSE assay revealed intra- and inter-assay variation of 4.9% and 10.3%, respectively. NSE was stable at room temperature for 4 h (*n* = 4, within subject CV = 8.0%) and for 24 h at 4 °C (*n* = 4, within subject CV = 11.2%). Sensitivity, defined as the readout of 3×s.d. (standard deviation) of low samples, was 62 pg/ml.

### Total grey matter volume

Among adults, we obtained 842 T1-weighted magnetic resonance imaging (MRI) scans with two 3T General Electric platforms: 411 scans were obtained using a GE 3T Signa HDxt scanner with an eight-channel head, and 431 scans using a GE 3T Discovery 750 scanner with a 32-channel head coil. Among adolescents, 85 T1-weighted MRI scans were obtained using the same scanners: 49 scans were acquired using the GE Signa HDxt system and 36 scans were acquired using the GE Discovery 750 system. The acquisition sequences used are described in online Supplementary material.

In order to obtain the TGMV, all MRI scans were processed using FreeSurfer v6.0 (Fischl, 2012). TGMV was calculated as the sum of the cerebral cortical volume, the subcortical grey matter and the cerebellum grey matter (https://surfer.nmr.mgh.harvard.edu/fswiki/MorphometryStats). Quality inspection and editing was performed by trained research assistants following standard FreeSurfer procedures (McCarthy et al., 2015).

### Statistics

All analyses were conducted separately for the adult and the adolescent samples. The distribution of the NSE concentrations was highly positively skewed in both samples, and we applied logarithmic transformations (log_10_) to normalise the data. The logNSE concentrations were approximately normally distributed in both samples (online Supplementary Figs S1 and S2). In the bivariate analysis, we assessed group differences between patients and HC in sex, age and body mass index (BMI) as well as the correlations among each of these variables and logNSE. In the patient group, we also investigated putative correlations between age of onset, duration of illness (DOI), PANSS total score, YMRS score and medication variables, and logNSE (Table 1). In our multivariate models, we investigated putative associations between disease status (patients *v.* HC) and logNSE concentrations, controlling for variables that were significantly correlated with logNSE in the bivariate analysis. For the main analyses, we computed partial eta-squared, one of the most largely used effect size belonging to the *r* family (Lakens, 2013). Partial eta-squared measures the strength of the patient/control-logNSE association and describes the proportion of variance in logNSE explained by the patient/control status. In adults, we also explored putative NSE level differences between patient subgroups (SZ spectrum and BD spectrum) and HC. In the TGMV analysis, we in addition controlled our multivariate models for TGMV and scanner.

**Table 1.** Group differences between adult patients with SMI and HC as well as adolescent patients with SMI and HC

	Patients with SMI		Healthy controls			Correlation with logNSE	
	*N*^a^	Mean (s.d.) or %	*N*^a^	Mean (s.d.) or %	*p* Value^b^	Direction (+ or −)	*p* Value^c^
Adult sample							
Sex (% females)	1132	48.3	903	45.6	0.226	−^d^	**<0.001**
Age (years)	1132	32.3 (10.7)	903	33.6 (9.2)	**0.004**	+	0.252
BMI (kg/m^2^)	1068	26.2 (5.3)	706	24.5 (3.7)	**<0.001**	+	0.354
TGMV (mm^3^)	333	689 048 (64 796)	509	699 739 (60 900)	**0.015**^e^	+	0.177
Age of onset (years)	1105	22.8 (8.4)				+	0.116
DOI (years)	1105	9.6 (9.1)				−	0.461
On antipsychotics (%)	1121	72				−	0.124
CPZ (mg/day)	781	313.5 (236.1)				+	**0.023**
On lithium (%)	1121	8.4				+	**0.003**^f^
On antiepileptics (%)	1121	20.5				+	0.504
On antidepressants (%)	1121	29.9				−	0.091
PANSS total score	1114	56.2 (16.5)				−	**0.049**
YMRS score	978	4.3 (4.9)				−	0.353
Adolescent sample							
Sex (% females)	32	62.5	67	52.24	0.337	−^d^	**0.025**
Age (years)	32	16.5 (1.3)	67	16 (1.4)	0.095	−	0.284
BMI (kg/m^2^)	27	23 (5.5)	54	21.1 (3.2)	0.225^g^	+	0.761
TGMV (mm^3^)	24	708 856 (58 658)	61	751 087 (69 388)	**0.010**^e^	−	0.489
Age of onset (years)	30	14.5 (2)				+	0.086
DOI (years)	30	1.1 (2.7)^h^				−	**0.021**
On antipsychotics (%)	32	59.4				+	0.793
CPZ (mg/day)	19	260.7 (163.2)				+	0.656
CPZ years	21	5.4 (27)^h^				−	0.268
PANSS total score	27	72.1 (16)				−	0.652
YMRS score	28	2.9 (3.8)				−	0.268

Group differences between adult patients with severe mental illness (SMI) and healthy controls (HC) as well as adolescent patients with SMI and HC in sex, age, body mass index (BMI) and total grey matter volume (TGMV). In patients, age of onset, duration of illness (DOI), Positive and Negative Syndrome Scale (PANSS) total score, Young Mania Rating Scale (YMRS) score, the percentage of patients on psychotropic medications as well as the chlorpromazine equivalent doses (CPZ) among patients on antipsychotics are presented. For adolescent patients, we also show lifetime CPZ (CPZ years). Two and one adolescent patients were on antidepressants and antiepileptics, respectively. Correlations with logNSE [log_10_ neuron-specific enolase (NSE)] are shown. *p* values <0.05 shown in bold.

a Number of participants with data in each variable.

b Chi-squared test or *t* test.

c Point-biserial correlation for binary variables; Spearman's correlation for continuous variables.

d Females had lower logNSE concentrations than males.

e Among adults but not adolescents, whilst controlling for age, sex and scanner, the case-control difference in TGMV was still significant (*p* < 0.001, and 0.157, respectively).

f Mann–Whitney *U* test confirmed that patients on lithium had significantly higher circulating NSE concentrations than patients not on lithium (*p* = 0.003).

g Mann–Whitney *U* test.

h Median (interquartile range).

## Results

### Main analysis

#### Adult sample

**Fig. 1.**
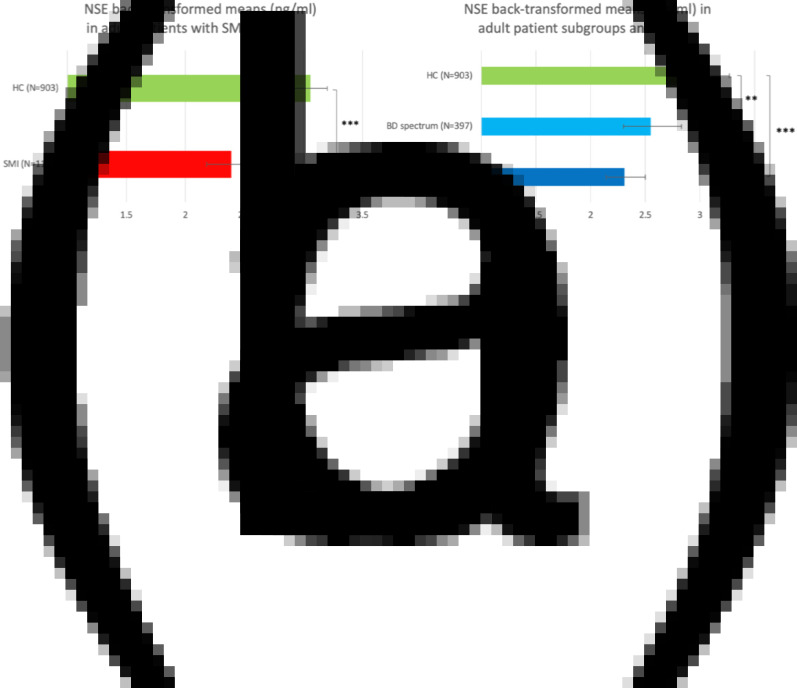
Back-transformed plasma NSE means in (a) adult patients with SMI and HC and (b) adult patients with SZ and BD and HC. **p = 0.005, ***p < 0.001.

Patients and HC differed significantly in age and BMI with lower mean age and higher mean BMI in the patients (Table 1). Sex, but not age (see online Supplementary Fig. S3 for visualisation of the lack of age-logNSE correlation) or BMI, was correlated with logNSE assessed with point-biserial correlation, *r*_pb_ *=* −0.117, *p* < 0.001, with lower logNSE in females than males, and sex was therefore included in the multivariate analysis. Among patients, the PANSS total score was inversely correlated with logNSE assessed with Spearman's correlation, *r*_s_ = −0.059, *p* = 0.049. In the multivariate model (analysis of variance, ANOVA), there was a highly significant main effect of disease status (SMI/HC) on logNSE, *F* = 27.296, *p* < 0.001, partial eta-squared (*η*^2^) = 0.013, with lower logNSE in patients than in HC (Fig. 1a). Sex had also a significant effect on logNSE, *F* = 27.305, *p* < 0.001, *η*^2^ = 0.013, with lower logNSE in women than men. The sex effect was also present when we stratified the analysis by disease status, such that both women with SMI had lower logNSE than men with SMI (*p* < 0.001) and healthy women had lower logNSE than healthy men (*p* = 0.011).

We separated the adult patients into SZ spectrum (*n* = 735) and BD spectrum (*n* = 397). The bivariate analysis showed that there were significant sex, age and BMI differences between SZ spectrum, BD spectrum and HC (online Supplementary Table S1). Only sex was correlated with logNSE (Table 1) and was accounted for in the multivariate analysis. In the multivariate model (ANOVA), there was a highly significant main effect of disease status on logNSE, *F* = 14.762, *p* < 0.001. We ran three pairwise comparisons and accepted statistical significance at a Bonferroni-adjusted alpha level of 0.017 (0.05/3). Pairwise comparisons showed that both patients with SZ spectrum and BD spectrum disorders had significantly lower logNSE than HC (*p* < 0.001 and *p* = 0.005, respectively), whereas there was no significant difference in logNSE between the two patient groups (*p* = 0.137) (Fig. 1b). There was still a significant main effect of sex on logNSE, *F* = 28.951, *p* < 0.001. Finally, we followed up the logNSE-PANSS correlation described in the previous paragraph. Stratifying by diagnostic group (SZ spectrum/BD spectrum) and controlling for sex, we ran two multiple linear regressions and showed that logNSE was significantly inversely associated with PANSS total score in SZ spectrum (*n* = 719, *β* = −0.105, *p* = 0.005), but not in BD spectrum (*n* = 395, *β* = −0.098, *p* = 0.059). Multiple linear regressions on PANSS positive, negative and general scores, adjusting for sex, showed that in SZ spectrum, logNSE was inversely associated with the positive (*b* = −0.092, *p* = 0.014), the general (*b* = −0.088, *p* = 0.019) but not the negative PANSS score, while in BD spectrum, with the positive score (*β* = −0.111, *p* = 0.029) only.

For both multivariate analyses, we also applied full factorial models where we included both factors and their interaction; there were no significant disease status-by-sex interactions (*p* = 0.185 and 0.112 for the interaction in SMI/HC and SZ spectrum/BD spectrum/HC analysis, respectively). As both age and BMI differed among the analysed groups (Table 1 and online Supplementary Table S1), we inserted both variables as covariates in both multivariate models. In the SMI/HC ANCOVA (analysis of covariance), the associations between disease status (*p* = 0.002) and sex (*p* < 0.001) with logNSE remained significant, whereas age (*p* = 0.665) and BMI (*p* = 0.547) were not significantly associated with logNSE in line with the results of the bivariate analysis. In the SZ spectrum/BD spectrum/HC ANCOVA, the corresponding *p*-values were 0.002, <0.001, 0.803, and 0.513.

#### Adolescent sample

**Fig. 2.**
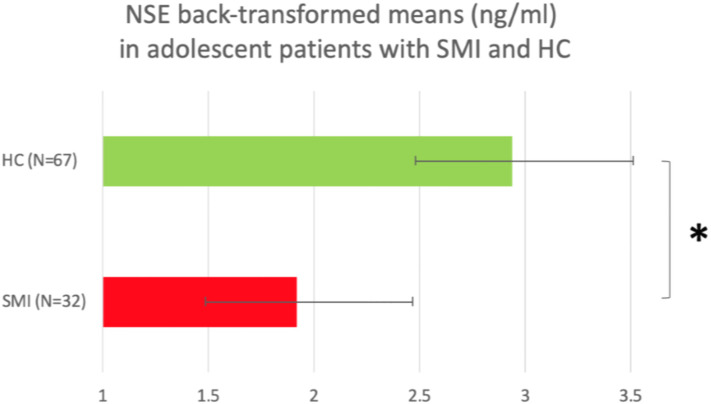
Back-transformed plasma NSE means in adolescent patients with SMI and adolescent HC. *p < 0.05.

Patients and HC did not significantly differ in sex, age or BMI (Table 1). As in adults, sex, but not age or BMI, was correlated with logNSE, *r*_pb_ *=* −0.225, *p* = 0.025, with lower logNSE in females than males, and sex was thereby included in the multivariate analysis. Among patients, DOI was inversely correlated with logNSE, *r*_s_ *=* −0.419, *p* = 0.021. In the multivariate model (ANOVA), there was a significant main effect of disease status on logNSE, *F* = 7.672, *p* = 0.007, *η*^2^ = 0.074, with lower logNSE in patients than in HC (Fig. 2). Also sex had a significant effect on logNSE, *F* = 4.318, *p* = 0.04, *η*^2^ = 0.043, with lower logNSE in females than in males. We finally applied a full factorial model showing no disease status-by-sex interaction (*p* = 0.416 for the interaction).

### Total grey matter volume analysis

We included 333 adult patients and 509 adult HC (adult MRI sample), and 24 adolescent patients and 61 adolescent HC (adolescent MRI sample). In both samples, females had smaller TGMV than males (*p* < 0.001 for both) assessed with *t* tests, while age was negatively correlated with TGMV assessed with Spearman's correlations (*p* < 0.001 and *p* = 0.005, respectively). Applying ANCOVAs controlling for sex, age and scanner, patients had lower TGMV than HC in the adult but not the adolescent sample (Table 1). TGMV was not correlated with logNSE in any sample (Table 1).

In order to evaluate a putative TGMV influence on our main results, we first repeated the main ANOVAs in our MRI samples, and subsequently added the TGMV and scanner variables and ran ANCOVAs. In adults, the disease status effect (*p* = 0.011 and 0.013 in the ANOVA and the ANCOVA, respectively) and the sex effect (*p* < 0.001 in both analyses) on logNSE were significant. In the adolescent sample, the corresponding *p*-values were 0.001, <0.001, 0.337 and 0.069.

### Medication and substance use analysis

Among adults, we restricted the analysis to patients not currently on any psychotropic medication (*n* = 156) and HC, and applied an ANOVA to determine main effects of disease status and sex on logNSE. Both disease status and sex had still significant main effects on logNSE, with lower logNSE in patients than HC (*p* = 0.004) and in women than men (*p* = 0.001). Among adult patients, the current use of lithium (*p* = 0.003) and the CPZ (*p* = 0.023) were positively correlated with logNSE. Among adolescent patients, neither current use of antipsychotics, CPZ nor CPZ years were significantly correlated with logNSE (Table 1).

Among adults, restricting the analysis to participants with a zero DUDIT score and controlling for the AUDIT score did not change the results (*p* < 0.001 for both disease status and sex effect on logNSE) (online Supplementary Material).

## Discussion

We showed that circulating NSE levels were significantly *lower* in adult and adolescent patients with SMI compared to HC, and in adults, the same pattern was seen in SZ and BD spectra. The results remained significant after controlling for TGMV, diminishing the possibility that the lower NSE levels in the patient groups reflect smaller TGMV.

CSF NSE concentrations in SZ have been investigated by others, but the results have been contradictory most probably due to the small sample size used (Egan et al., 1992; Li, Wu, Guo, & Zhao, 2006; Steiner et al., 2006; Vermuyten, Lowenthal, & Karcher, 1990). Circulating NSE has been explored in SZ in studies of small sample sizes (25 to 39 participants) all showing the lack of significant NSE difference between patients and HC (Egan et al., 1992; Schroeter et al., 2009; Steiner et al., 2006). Circulating NSE levels have also been explored in BD with conflicting results with a recent meta-analysis reporting the lack of association with the illness (Bartoli, Misiak, Crocamo, & Carra, 2020). In line with our results, Wiener et al. reported lower serum NSE levels in unmedicated patients with BD (*n* = 36, mean age 28 years) compared to HC (*n* = 36, mean age 28 years) (Wiener et al., 2013). Machado-Vieira et al. showed lower plasma levels in both unmedicated (*n* = 30, mean age 26 years) and lithium-treated patients (*n* = 15, mean age 26 years) with BD during a manic episode relative to HC (*n* = 30, age matched) (Machado-Vieira et al., 2007). Akcan et al. reported that patients with chronic (*n* = 22, mean age 29 years) BD had lower serum NSE levels than first episode (*n* = 24, mean age 25 years) BD and HC (*n* = 19, mean age 25 years) (Akcan et al., 2018). By contrast, Karabulut et al. reported higher plasma NSE in patients with chronic BD (*n* = 77, mean age 38 years) compared to patients with early stage BD (*n* = 30, mean age 25 years) and HC (*n* = 30, mean age 32 years) (Karabulut et al., 2019). Finally, Tsai et al. reported no serum NSE level difference between patients with BD in a manic state (*n* = 17, mean age 37 years) and HC (*n* = 30, mean age 34 years), and among patients no changes in NSE levels after treatment (Tsai & Huang, 2017).

The discrepancy between our results and some of the previous studies on circulating NSE might be due to the large differences in sample sizes. None of the previous original studies included more than 150 participants whereas we investigated 1132 adult patients with SMI and 903 adult HC as well as 32 adolescent patients with SMI and 67 adolescent HC yielding a substantial increase in power and the advantage of using two independent samples representing different ages. Interestingly, the meta-analysis in BD included 251 patients and 145 HC, and showed non-significantly lower circulating NSE levels in patients with high heterogeneity across studies (Bartoli et al., 2020). Further, NSE is present in platelets in much lower but not negligible levels compared to the cortical brain tissue (Marangos, Campbell, Schmechel, Murphy, & Goodwin, 1980a), and we cannot exclude that the use of serum *v.* plasma as in the present study could have contributed to the discrepancy due to the release of platelet components such as NSE during coagulation of serum *in vitro* (Yu et al., 2011).

We found significantly lower NSE levels not only in adult but also in adolescent patients with SMI. Up to 18% of patients with psychosis develop their first episode before reaching adulthood, and they may be at higher risk of poor outcome relative to the patient group with the adult-onset type of the disorder (Diaz-Caneja et al., 2015). In addition to replicating the result from the much larger adult sample, we can show that the lower plasma NSE concentrations in patients with SMI are present already in adolescence close to the disease onset (median DOI 1 year). This might support the notion that the lower NSE levels are related to an underlying disease mechanism rather than being an epiphenomenon of longstanding disease. Furthermore, the patient/control status–NSE association was stronger in adolescents with 7.4% of the variance in logNSE explained by the patient-control status *v.* 1.3% in adults, which may further support the notion of NSE involvement in neurodevelopment. Adolescence represents the second period of major neurodevelopment with abundant ongoing maturation processes (Arain et al., 2013) and may be a window of increased susceptibility to neurodevelopmental disturbances (Brent et al., 2013). Among adolescent patients, the DOI was inversely correlated with NSE which may reflect this late neurodevelopmental disturbance with decreasing NSE levels as the disorder progresses. Among adult patients with SZ spectrum disorders, NSE levels were inversely associated with PANSS total, positive and general score, while among adult patients with BD spectrum disorders with PANSS positive score. These observations raise the possibility that lower NSE might be related to a more severe form of SMI.

Among adults and adolescents, females (patients and HC combined) had significantly lower circulating NSE than males. In adults, this difference was present in both HC and patients. A recent study investigating serum NSE in over 10 000 healthy adults (20–79 years) reported no significant difference between men and women (Liu et al., 2019). Another study investigating 901 healthy individuals reported a sex-by-age interaction. In particular, women had lower serum NSE levels compared to men in younger ages (<60 years) and higher in older ages (Hoffmann et al., 2017). This is partially in line with our result showing lower NSE levels in healthy adult women than in healthy men (age range 18–65 years). We did not find sex-by-age interactions on logNSE in patients and HC combined, patients or HC (online Supplementary Material). In line with our finding, the female brain has been shown to have lower metabolic age indicating preservation of developmental attributes (Goyal et al., 2019).

We found that the subgroup of adult patients who currently did not use psychotropic medication still had significantly lower NSE levels than HC indicating that the NSE level differences found in our main analyses were not confounded by psychotropic medication use in patients. Further, among adult patients the antipsychotic dosage as well as the use of lithium were both positively correlated with NSE levels. We are thereby tempted to speculate that antipsychotics and lithium ameliorate the SMI-related maturation disturbance. This notion is supported by animal studies showing that antipsychotics enhance differentiation and maturation in dentate gyrus (Chen et al., 2013) and oligodendrocyte precursor cells (Xu, Yang, & Li, 2014) while lithium has been shown to enhance the differentiation of neural progenitor cells (Su, Chu, & Wu, 2007; Su et al., 2009).

Our results, indicating a lack of progressive neuronal damage, are in line with other reports indicating an absence of neurodegeneration in SMI (Barth et al., 2020; Haukvik et al., 2016; Zipursky, Reilly, & Murray, 2013). The neurodevelopmental model has mainly been discussed for SZ (Kahn et al., 2015; Zipursky et al., 2013), whereas in BD a neurodevelopmental pathogenesis is more controversial (Kloiber et al., 2020). Our results can be interpreted as an additional indication of neurodevelopmental origin and lack of substantial neurodegeneration in both disorders. Although there may be different underlying mechanisms across and within diagnostic categories, the findings are in line with the clinical observation that patients with SMI may achieve long-term stability and to some extent even recovery. Of note, whereas the lack of knowledge as well as attribution to genetics may increase stigmatising attitudes towards patients (Hawke et al., 2013; Serafini et al., 2011; Valery & Prouteau, 2020), recovery-oriented strategies can facilitate destigmatisation (Valery & Prouteau, 2020).

The present study has certain limitations. Due to the multiple and partially unknown role of NSE (Haque et al., 2018; Isgro et al., 2015), we cannot rule out alternative interpretations of the reduced NSE levels in SMI. NSE is a glycolytic enzyme (Isgro et al., 2015) and its reduced circulating levels may reflect impaired neuronal glycolytic capacity in SMI. NSE is also present in the cytoplasm of central and peripheral neuroendocrine cells (Marangos & Schmechel, 1987) and is elevated in patients with neuroendocrine tumours (Isgro et al., 2015). Thus, we cannot rule out that the observed lower NSE levels in SMI reflect dysfunction within neuronal glycolysis or the neuroendocrine system. Another limitation of the study is the inclusion of both inpatients and outpatients diagnosed with SZ or BD spectrum disorders. We note, however, that when the two diagnostic categories were analysed separately, both showed significantly lower NSE levels than HC. Moreover, although the overall number of participants was high, the number of adolescent patients was rather low. Further, as the distribution of the NSE concentrations in both adolescents and adults was highly positively skewed, we used the log_10_-transformed NSE variable, which may have implications for the interpretation of the results (Feng et al., 2014). To deal with this limitation, we ran sensitivity analyses with nonparametric statistics, where we used the original NSE variable, and confirmed the main results showing significantly lower NSE concentrations in adults (both for SZ and BD spectrum) and adolescents with SMI compared with HC (online Supplementary Material). Finally, we cannot exclude that a portion of the measured NSE originates from platelet-stored NSE (Marangos et al., 1980a) release during freeze and thaw cycles. However, we believe it is unlikely that there is a differential platelet-stored NSE release in patients and HC that can generate the observed case-control plasma NSE difference.

To conclude, we found significantly lower circulating NSE concentrations in both adult and adolescent patients with SMI compared to HC. As an elevated NSE is considered to reflect neuronal injury, whereas lower NSE may index neuronal maturation disturbances, our results support the notion of abnormal neurodevelopmental rather than neurodegenerative processes in SMI.

## Supplementary material

For supplementary material accompanying this paper visit https://doi.org/10.1017/S0033291721003056. click here to view supplementary material
