# Supplementary material for: Lower circulating neuron-specific enolase concentrations in adults and adolescents with severe mental illness
Source: Psychol Med. 2021 Aug 11;53(4):1479–88. doi: 10.1017/S0033291721003056 (PMC10009386; doi:10.1017/S0033291721003056)
Supplement: Supplementary file 1 [file S0033291721003056sup001.docx]

**Supplementary material**

**Description of T1-weighted MRI sequences**

411 scans in adults and 49 scans in adolescents were obtained using a GE 3T Signa HDxt scanner with an 8-channel head coil. For these, a 3D fast spoiled gradient echo (FSPGR) sequence was acquired using the following parameters: 170 sagittal slices, slice thickness = 1.2 mm, voxel size = 1x1x1.2 mm, inversion time (TI) = 450 ms, echo time (TE) = MinFull, repetition time (RT) = 7.8 ms, flip angle = 12°. 431 scans in adults and 36 scans in adolescents were obtained using a GE 3T Discovery 750 scanner with a 32-channel head coil. For these, a 3D ultrafast gradient echo (BRAVO) sequence was acquired using the following parameters: 188 sagittal slices, slice thickness = 1 mm, voxel size = 1x1x1 mm, TI = 450 ms, TE = 3.18 ms, TR = 8.16 ms, flip angle = 12°.

**Supplementary results**


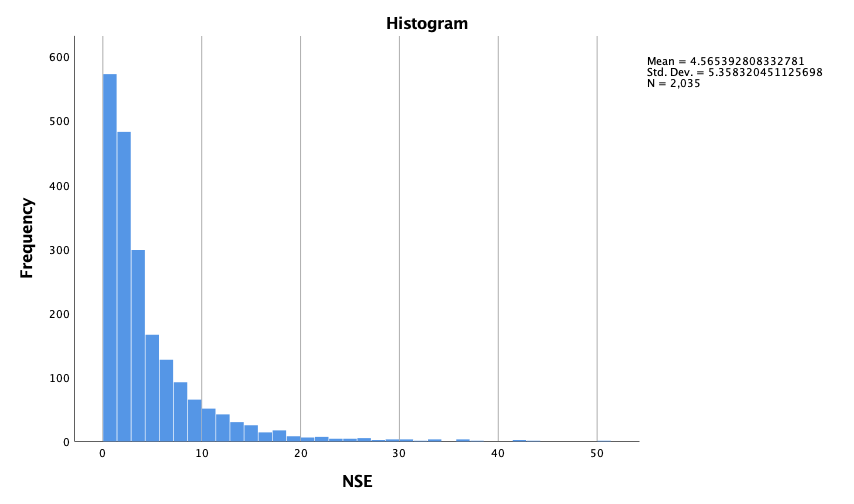
**
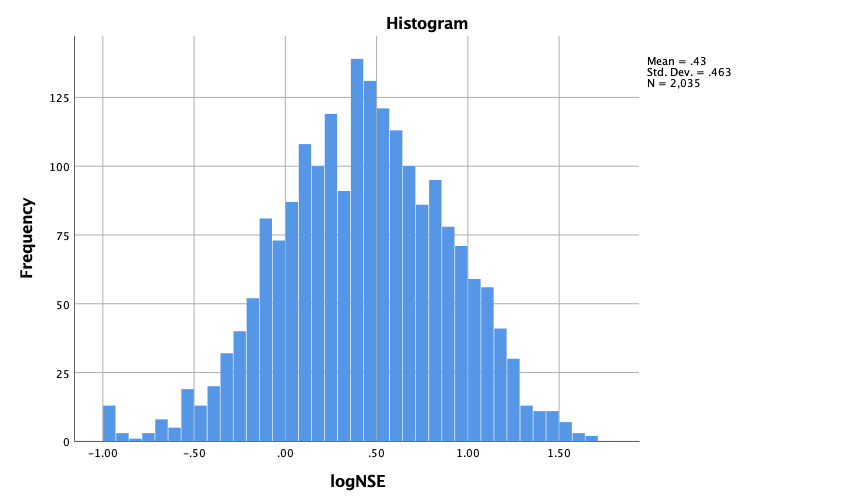
**

**Supplementary Figure 1**. Distribution of plasma neuron-specific enolase (NSE) (ng/ml) and logNSE concentrations in the adult sample. The distribution of logNSE was approximately normally distributed as evaluated by visual inspection


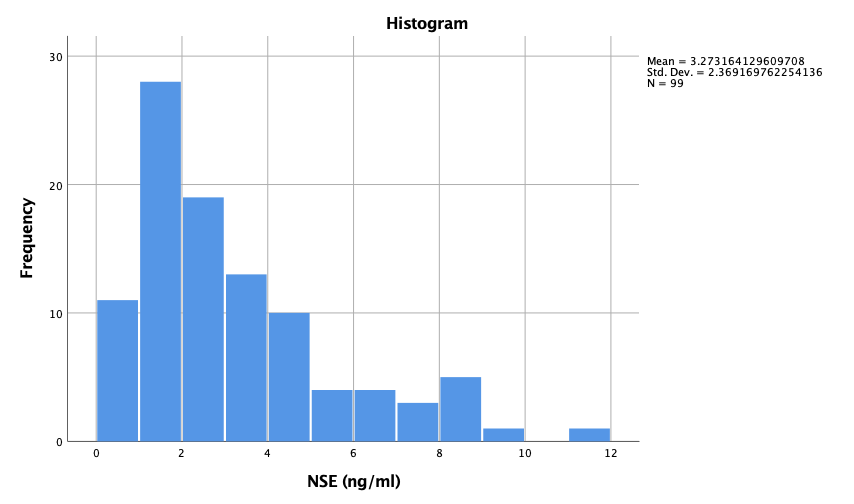

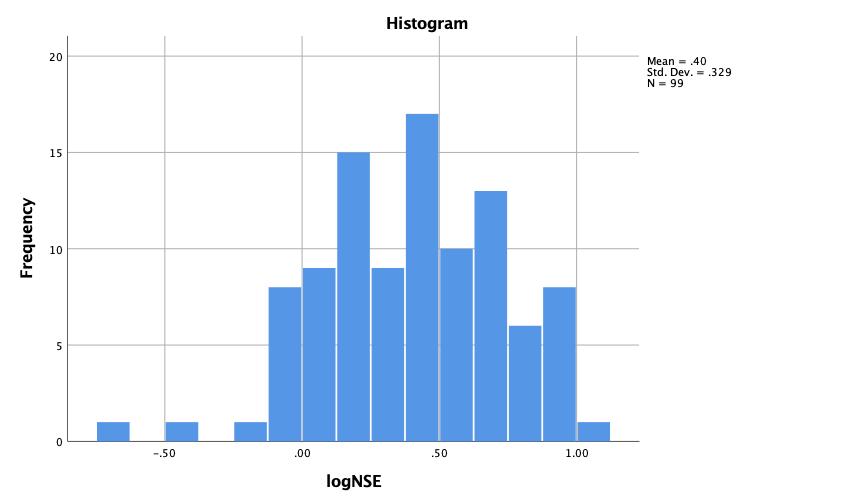


**Supplementary Figure 2**. Distribution of plasma neuron-specific enolase (NSE) (ng/ml) and logNSE concentrations in the adolescent sample. The distribution of logNSE did not significantly deviate from normality assessed by the Shapiro-Wilk test of normality (*p*=0.231)

***Supplementary results in adults***

*SMI/HC analysis*

In the multivariate model applied in SMI/HC, the back-transformed adjusted marginal NSE means were 2.39 (95% CI, 2.25 to 2.55) and 3.06 (95% CI, 2.86 to 3.27) ng/ml for patients and HC, respectively (Figure 1A). There was homogeneity of variances evaluated with Levene’s test, *p*=0.595. The residuals of the overall model were approximately normally distributed determined by visual inspection. Observations with studentized residuals greater than 3 in absolute value were considered outliers. There were 11 outliers with studentized residuals less than -3 standard deviations. The cases did not exhibit high leverage or high Cook's distance value and were included in the final model presented in the main text. Exclusion of these cases did not change any results.

We also inserted age and the interaction term sex-by-age in the multivariate model in SMI/HC. There was no sex-by-age interactive effect on logNSE (*p*=0.917). In addition, we analyzed separately patients with SMI and HC and also failed to find sex-by-age interactive effect on logNSE (*p*=0.593 and 0.522, respectively).

**
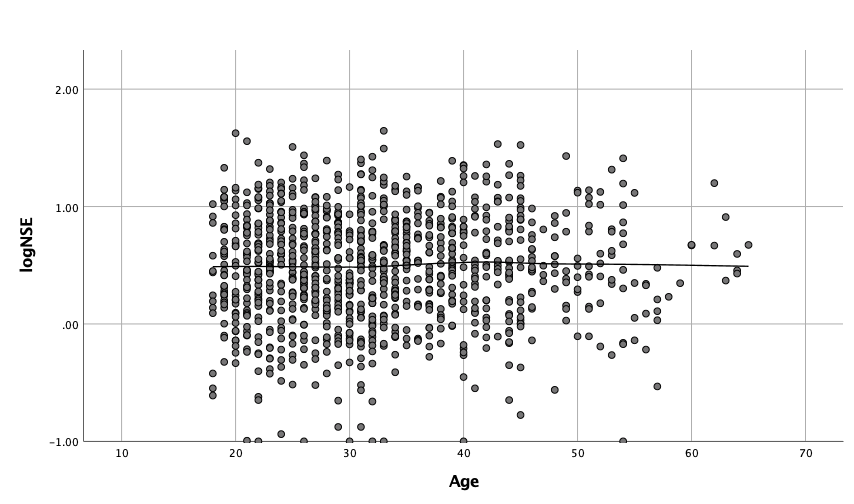

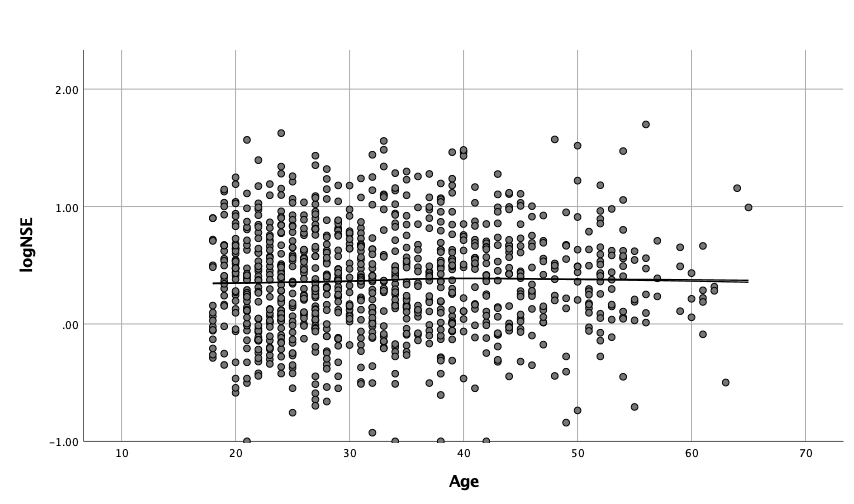
**

**Supplementary Figure 3.** Scatterplots of logNSE and age in male (left) and female (right) adult participants. Fit method: Loess line, 70% of points to fit

*SZ spectrum/BD spectrum/HC analysis*

In the multivariate model applied in SZ spectrum/BD spectrum/HC analysis, the back-transformed adjusted marginal NSE means were 2.31 (95% CI, 2.14 to 2.5) , 2.55 (95% CI, 2.3 to 2.83) and 3.06 (95% CI, 2.86 to 3.27) ng/ml for SZ spectrum, BD spectrum and HC, respectively (Figure 1B). There was homogeneity of variances evaluated with Levene’s test, *p*=0.453. The residuals of the overall model were approximately normally distributed determined by visual inspection. There were 10 outliers with studentized residual less than -3 standard deviations. The cases did not exhibit high leverage or high Cook's distance value and were included in the final model presented in the main text. Exclusion of these cases did not change any results.

|  | SZ spectrum | | BD spectrum | | HC | |  |
| --- | --- | --- | --- | --- | --- | --- | --- |
| Adults | N | Mean (SD) or % | N | Mean (SD) or % | N | Mean (SD) or % | P value^1^ |
| Sex (% women) | 735 | 41.6 | 397 | 60.7 | 903 | 45.6 | **<0.001** |
| Age (years) | 735 | 31.2 (9.9) | 397 | 34.2 (11.8) | 903 | 33.6 (9.2) | **<0.001** |
| BMI (kg/m^2^) | 685 | 26.3 (5.4) | 383 | 26 (5.1) | 706 | 24.5 (3.7) | **<0.001** |

**Supplementary Table 1**. Group differences between patients with schizophrenia (SZ) spectrum disorders, bipolar (BD) spectrum disorders and healthy controls (HC) in sex, age and body mass index (BMI).

^1^Chi-square test for sex; Welch ANOVA for age and BMI

*Substance use analysis*

Among adults, we had data on AUDIT score for 862 patients and 635 HC. Patients and HC did not differ in AUDIT score assessed with Mann-Whitney U test (*p*=0.156); AUDIT score was not significantly correlated with logNSE, *r_s_*=0.049, *p*=0.059. DUDIT scores were available for 916 patients and 634 HC of which 600 patients and 587 HC had a DUDIT score of zero. We restricted the analysis to participants with a zero DUDIT score and applied an ANOVA to determine main effects of disease status and sex on logNSE. There was still a significant effect of disease status on logNSE (*p*<0.001), with lower logNSE in patients, and a significant effect of sex on logNSE (*p*<0.001) with lower logNSE in women. Applying an ANCOVA controlling for the AUDIT score did not change the results (p<0.001 for both disease status and sex effect).

***Supplementary results in adolescents***

In the small adolescent sample, there was one participant (female patient with psychosis NOS) with an extremely high NSE value (16.618 ng/ml, > 3×interquartile range above the third quartile) (Suppl. Figure 4). In the multivariate model, there was one participant (male HC) with studentized residual less than -3 standard deviations. Both cases were excluded from the analysis reported in the main text. In a multivariate model including the two cases, there was a significant main effect of disease status on logNSE, *F*(1,98)=4.036, *p*=0.047, such that patients had lower logNSE than HC. Males had higher NSE concentrations than females, though the difference was not statistically significant, *F*(1,98)=1.962, *p*=0.164.

In the analysis reported in the main text, the back-transformed adjusted marginal NSE means were 1.92 (95% CI, 1.49 to. 2.47) and 2.94 (95% CI, 2.48 to 3.51) for patients and HC, respectively (Figure 2). Homogeneity of variances was assessed with Levene’s test, *p*=0.523. The residuals of the overall model were approximately normally distributed determined by Shapiro-Wilk test of normality (*p*=0.231).


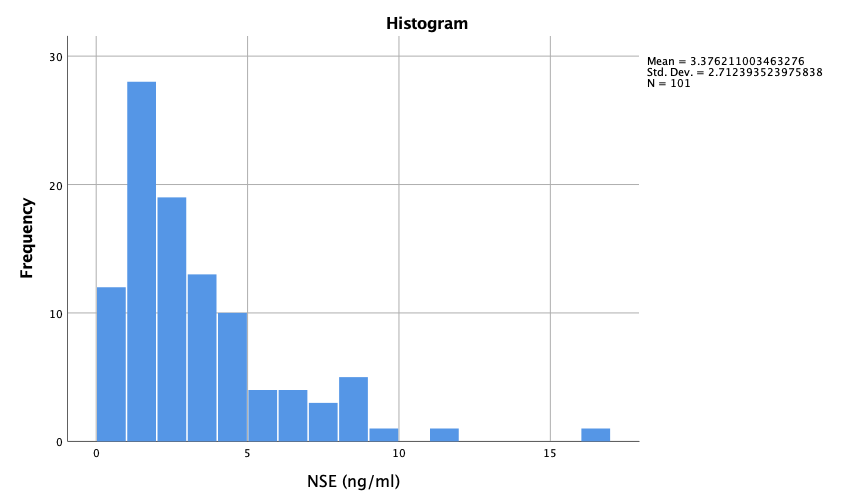

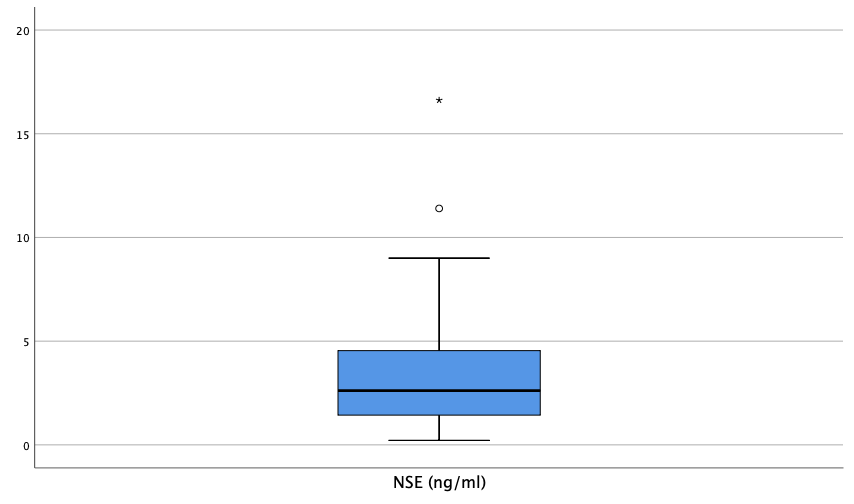


**Supplementary Figure 4.** Figures of the distribution of neuron-specific enolase (NSE) in the adolescent sample showing the extreme outlier (*)

***Sensitivity analysis***

Among adults, we ran a Mann-Whitney U test which confirmed the main result of the SMI/HC analysis; median NSE was statistically significantly lower in patients with SMI (2.49 ng/ml) than in HC (3.1 ng/ml) (p<0.001). Among adults, we also ran a Kruskal-Wallis test which confirmed the main result of the SZ spectrum/BD spectrum/HC analysis; both patients with SZ spectrum (2.38 ng/ml) and BD spectrum (2.66 ng/ml) disorders had significantly lower median NSE than HC (3.1 ng/ml) (p<0.001 and *p*=0.005, respectively), whereas there was no difference in median NSE between the two patient groups (*p*=1.000). Finally, among adolescents, we ran a Mann-Whitney U test which confirmed the main result; median NSE was statistically significantly lower in patients with SMI (1.81 ng/ml) than in HC (3.11 ng/ml) (*p*=0.007).

***NSE on hippocampal and amygdalar volumes***

We investigated for associations between logNSE concentrations and amygdala and hippocampus volumes, and we have not found evidence for such associations in any of the studied groups. In particular, applying age-, sex-, ICV-, and scanner-adjusted general linear models in the entire adult MRI sample (n=842), we found no associations between logNSE and left (*p*=0.818) or right (*p*=0.197) hippocampus or left (*p*=0.812) or right (*p*=0.272) amygdala volumes. The corresponding p-values when we stratified the analysis by patient/control status were 0.899, 0.279, 0.725 and 0.625 among patients with SMI (n=333), and 0.583, 0.828, 0.777 and 0,577 among HC (n=509). The corresponding p-values for a) the entire adolescent MRI sample (n=85), b) among adolescent patients with SMI (n=24) and c) among adolescent HC (n=61) were a) 0.463, 0.120, 0.328 and 0.367, b) 0.749,0.586, 0.450, 0.247 and c) 0.478, 0.327, 0.100, 0.348.
